# Generalized anxiety disorder in Berlin school children after the third COVID-19 wave in Germany: a cohort study between June and September 2021

**DOI:** 10.1186/s13034-022-00552-0

**Published:** 2023-01-03

**Authors:** Stefanie Theuring, Mascha Kern, Franziska Hommes, Marcus A. Mall, Joachim Seybold, Frank P. Mockenhaupt, Toivo Glatz, Tobias Kurth

**Affiliations:** 1 Institute of International Health, Charité - Universitätsmedizin Berlin, corporate member of Freie Universität Berlin and Humboldt-Universität zu Berlin, Augustenburger Platz 1, 13353 Berlin, Germany; 2 Institute of Public Health, Charité - Universitätsmedizin Berlin, corporate member of Freie Universität Berlin and Humboldt-Universität zu Berlin, Charitéplatz 1, 10117 Berlin, Germany; 3 Department of Pediatric Respiratory Medicine, Immunology and Critical Care Medicine, Charité - Universitätsmedizin Berlin, corporate member of Freie Universität Berlin and Humboldt-Universität zu Berlin, Augustenburger Platz 1, 13353 Berlin, Germany; 4 Medical Directorate, Charité - Universitätsmedizin Berlin, corporate member of Freie Universität Berlin and Humboldt- Universität zu Berlin, Charitéplatz 1, 10117 Berlin, Germany

**Keywords:** COVID-19, GAD-7, Anxiety, School children, Adolescents, Mental health, Germany

## Abstract

**Background:**

During the COVID-19 pandemic, children and adolescents worldwide have disproportionally been affected in their psychological health and wellbeing. We conducted a cohort study among German school children, aiming at assessing levels of general anxiety disorder (GAD) and identifying associated factors in the second pandemic year.

**Methods:**

A cohort of 660 students from 24 Berlin schools was recruited to fill in questionnaires including the GAD-7 tool on anxiety symptoms at three time points between June and September 2021. To adjust for non-random attrition, we applied inverse probability weighting. We describe reported GAD levels stratified by time point, sex, and school type and report odds ratios from univariate logistic regression.

**Results:**

In total, 551 participants (83%) filled in at least one questionnaire at any time point. At the first time point in June 2021, 25% of the children and adolescents reported anxiety symptoms with a GAD-7 score ≥ 5, decreasing to 16% in August 2021 directly after the summer holidays and rising again to 26% in September 2021. The majority of reported anxiety levels belonged to the least severe category. Being female, attending secondary school, coming from a household with lower education or with lower income level, and being vaccinated against COVID-19 were significantly linked with reporting anxiety symptoms. Preceding COVID-19 infection and anxiety were negatively associated.

**Conclusion:**

Overall, anxiety in school children was lower in mid-2021 than in the first pandemic year, but still double compared to pre-pandemic data. Reporting of anxiety symptoms during the second pandemic year was especially high in females and in secondary school students. Policy makers should pay additional attention to the mental health status of school children, even as the pandemic situation might stabilize.

## Introduction

Since the beginning of the worldwide spread of the novel coronavirus disease (COVID-19) in early 2020, it has affected children and adolescents far less than adults in terms of severe physical illness [1]. At the same time, the school-aged population has experienced a disproportional impact on psychological health and wellbeing because of disruptions of daily routine and social deprivation as a result of school closures [2–4].

Mental health needs among children and adolescents deserve utmost attention. Most mental disorders begin in childhood or youth, with a median age of onset as young as 11 years for anxiety or impulse-control disorders [5], and they will inevitably cause adverse social and health-related outcomes if early care and treatment are not provided [5, 6]. This is even more alarming given that pandemics such as COVID-19 have been found to not only be a precipitating cause of mental health decline [7], but also to exacerbate pre-existing mental health problems among children and adolescents [3, 7]. Recent research, including a global meta-analysis, revealed that during the COVID-19 pandemic, clinically significant generalized anxiety disorder (GAD) and depression in adolescents doubled compared to pre-pandemic times [8, 9]. A German study based on medical records from 168 pediatric practices suggests that for anxiety disorder and depression, the total number of children and adolescents diagnosed as well as the number of children and adolescents newly diagnosed increased by 9–13% in 2020 compared to the preceding year, with girls being more affected than boys [10]. However, the majority of related research focused on mental health impact during the initial pandemic stage, and there is a lack of data investigating long-term mental health developments in school-aged children over the course of the pandemic.

Between June 2020 and March 2021, the Berlin Corona School Study (BECOSS 1) investigated infection and transmission dynamics along with sociodemographic, behavioral and psychosocial parameters among school children, educational staff and connected household members in Berlin, Germany [11–13]. In a subsequent research phase (BECOSS 2), we assessed psychosocial wellbeing of primary and secondary school students during the later stage of the COVID-19 pandemic.

Here, we describe the presence of GAD among children and adolescents attending public schools in Berlin, Germany in June, August and September 2021, during a dynamic pandemic phase with alternating schooling and testing regimes and a holiday period.

## Methods

### Study setting

BECOSS 2 was conducted as a cohort study among school children in Berlin, Germany with three observation time points. To acquire a representative sample of public schools of Berlin, the 12 city districts were divided into three socio-economic strata, including “low”, “middle” and “high” [14]; per stratum, two districts were selected at random, yielding six districts. With the aim of selecting two primary and two secondary schools per district, all schools from the selected districts were put in random order and approached in that sequence. If schools rejected to participate, the next school on the list was approached until there was a total sample of 24 schools. Each school administration then chose classes to which the participation in the study was offered, depending on factors like class teachers' willingness and availability with regard to exams or school trips during the study period.

**Fig. 1:**
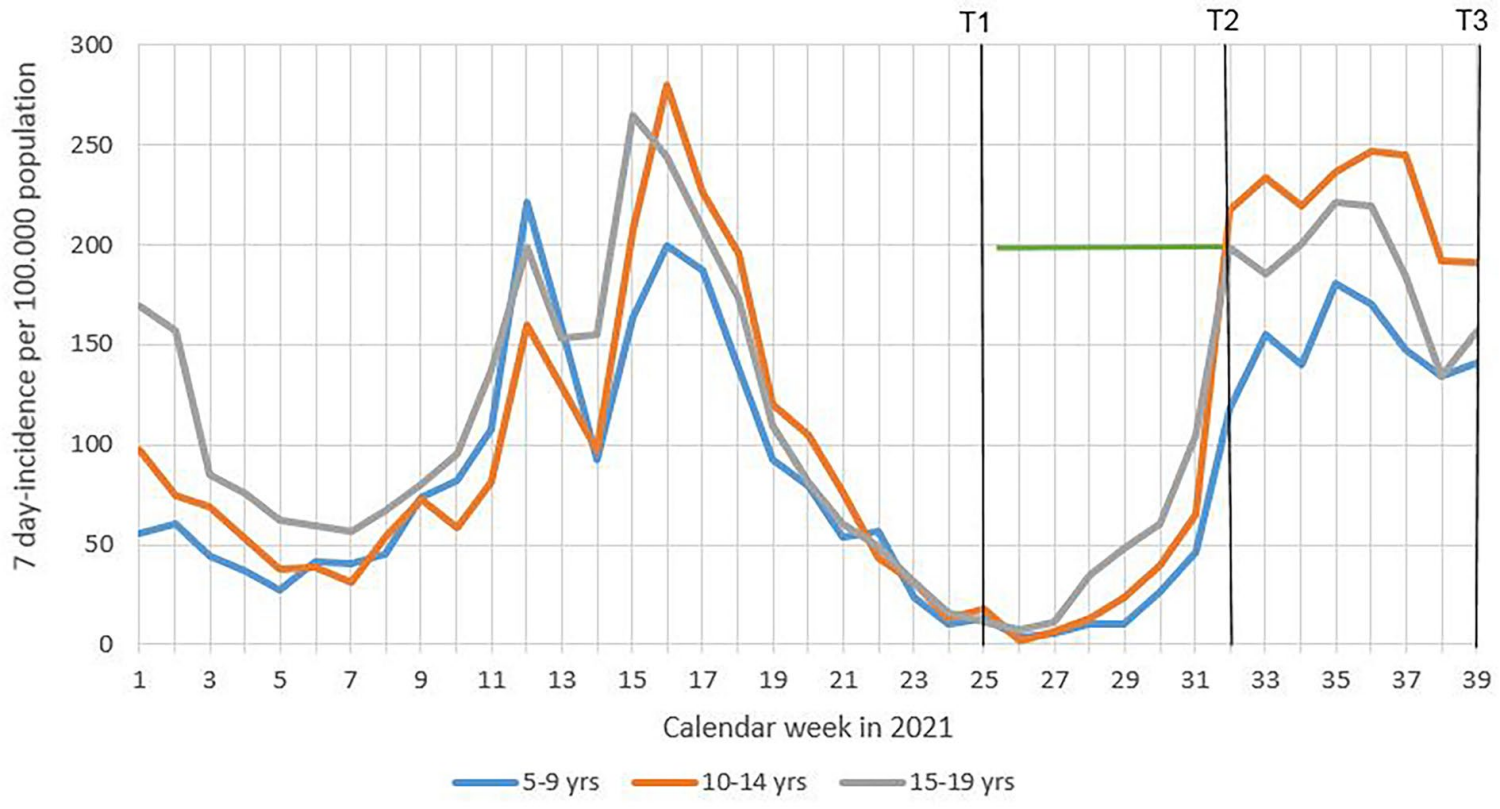
School-aged 7-day community incidence of SARS-CoV-2 in Berlin, 2021, study time points (black bars), and summer holidays (green line)

The three study time points included June 21–23, 2021 (T1), August 9–11, 2021 (T2), and September 27–29, 2021 (T3). In Berlin, a lockdown including school closure had been in effect between mid-December 2020 and mid-March 2021, i.e., before data collection for BECOSS 2 began. Observation points T1 and T2 took place directly before and after the summer holiday break (June 24–August 8). At T1, schools had resumed post-lockdown classroom teaching, with split classes at half of the original size and students attending school on alternate weeks. At T2, after the summer break, normal classroom teaching in the complete classes took place; school children and staff underwent obligatory, school-based SARS-CoV-2 rapid testing three times a week for the first three weeks of the school year. Additionally, they were obliged to wear masks. T3 at the end of September took place six weeks after students had resumed their usual school routine. At this time, wearing masks inside buildings was still obligatory, but SARS-CoV-2 testing was reduced to two times a week. Study time points are illustrated in Fig. 1.

### Data collection

Study information and consent forms were sent to the participating schools and distributed to the classes selected by the school administration. Consent forms were filled in by students who were willing to participate and their parents and submitted to the study team before the start of data collection. We received consent forms from 660 children in total. At each study time point, paper-based questionnaires were sent to the enrolled participant's home address with directions to fill them in. We asked parents to help their children to fill in the questionnaire for primary school children. After filling them in, at a pre-specified date for each study round, participants dropped the questionnaires off at a locked collection box at their school, which was picked up by the study team on the same day. Questionnaires were then digitized using FormPro Software (OCR Systems, Version 3.1) and transformed into an Excel database for further analysis.

Collected data included general sociodemographic and the family’s economic background information. Age and school type were assessed separately because in the Berlin school system, between the age period of approximately 10–14 years, children could be in primary or secondary school. Family migration background was defined as at least one parent not born in Germany. We doubled the average monthly net income of a Berlin household with one working adult in 2021 to define a “higher income” category as > 5.000€ [15]. General anxiety symptoms were assessed using the GAD-7 questionnaire [16, 17]. Study participants were asked about the presence of seven anxiety symptoms during the last two weeks. Response options included “not at all”, “on several days”, “more than half the days”, and “nearly every day,” which received points of 0, 1, 2, and 3, respectively. Consequently, individual anxiety scores ranged between 0 and 21. A GAD-7 score of ≥ 5 was defined as presence of anxiety symptoms. Depending on the score, the severity of anxiety was considered mild (5–9), moderate (10–14) or severe (15–21) [18].

### Data analysis

To correct for nonrandom attrition and non-response bias, we used an inverse probability weighting (IPW) approach [19]. As a first step, children were inversely weighted at each time point separately based on their age, sex and participation at previous time points (for T2 and T3). In a second step, these weights were multiplied across all time points yielding an IPW-population, referred to as pseudo-population, based on the weighted responses of those children that completed all three time points. This pseudo-population was used in all analyses.

Data analyses were conducted with R 4.1.3 [20]. We computed frequencies and percentages of GAD-7 outcomes stratified by time point, sex and school type. In addition, we estimated odds ratios for a binary GAD-7 outcome (presence of reported anxiety symptoms, GAD ≥ 5; vs. no reported anxiety symptoms, GAD < 5) using separate univariate logistic regression models for the independent variables chosen based on prior evidence [15, 21, 22]. The variables included age (one year increase), sex (female vs. male), school type (secondary vs. primary), socioeconomic status (mid vs. high and low vs. high), monthly household income (lower vs. higher), household education (secondary vs. higher education), family migration background (yes vs. no), vaccination status (at least one vaccination received vs. none received) and prior COVID-19 infection (at least one vs. none).

## Results

Out of the 660 enrolled students, a total of 480 students (73%) responded at T1, 489 students (74%) at T2, and 377 students (57%) at T3. In total, 551 participants (83%) filled in at least one questionnaire at any time point and 319 students (48%) filled in the questionnaire at all three time points. By means of IPW, these children with complete observations were weighted to count for the missing data of children of the same age and sex, resulting in a pseudo-population of 654 students, which is very close to the complete sample (see Table 1). Mean age at study start was 12.8 years (range 6–19 years).

**Table 1:** Characteristics of the inverse probability-weighted population at all three time points

Participants	*N*	T1	T2	T3
		654	654	654
Sex	Male Female	321 (49.1%) 333 (50.9%)	321 (49.1%) 333 (50.9%)	321 (49.1%) 333 (50.9%)
Age	Mean SD Range	12.8 2.3 6–19	13.0 2.3 6–19	13.1 2.3 7–19
School^a^	Primary Secondary	243 (37.1%) 412 (62.9%)	243 (37.1%) 412 (62.9%)	243 (37.1%) 412 (62.9%)
School district SES	High Middle Low	390 (59.6%) 155 (23.7%) 109 (16.6%)	390 (59.6%) 155 (23.7%) 109 (16.6%)	390 (59.6%) 155 (23.7%) 109 (16.6%)
Monthly household income	Higher Lower *N* missing	333 (52.6%) 300 (47.4%) 22	332 (52.5%) 300 (47.5%) 22	342 (54.0%) 291 (46.0%) 22
Household education	Higher Secondary *N* missing	571 (87.6%) 81 (12.4%) 2	585 (89.8%) 66 (10.2%) 2	571 (87.7%) 80 (12.3%) 2
Family migration background	Yes No *N* missing	120 (19.6%) 493 (80.4%) 42	140 (21.7%) 507 (78.3%) 6	142 (22.1%) 501 (77.9%) 10
Vaccination	Any None *N* missing	28 (4.3%) 612 (95.7%) 15	143 (21.9%) 511 (78.1%) 0	304 (46.5%) 350 (53.5%) 0
COVID-19 infection	Ever Never	35 (5.3%) 619 (94.7%)	41 (6.3%) 613 (93.7%)	43 (6.6%) 611 (93.4%)

*SD* standard deviation

^a^Age range is 6–14 years for primary schools and 10–19 years for secondary schools. Due to rounding of non-integer participant weights, the sum of individual strata might be ± 1 off from the total number of participants

Presentation of anxiety symptoms in the spectrum from mild to severe differed among time points with 24.8% of children reporting any anxiety symptoms just before the summer holidays (T1), decreasing to 16.2% directly after the summer holidays (T2) before going back up to 25.7% 6 weeks into the new school term (T3). Moderate or severe anxiety were reported in 4.8% at T1, 3.2% at T2 and 4.4% at T3 (Table 2).

**Table 2:** GAD-7 categories of anxiety symptoms per time point in the inverse probability-weighted population

GAD-7	T1	T2	T3
None	476 (75.2%)	541 (83.8%)	475 (74.3%)
Mild	126 (20.0%)	84 (13.0%)	136 (21.3%)
Moderate	21 (3.2%)	15 (2.3%)	24 (3.8%)
Severe	10 (1.6%)	6 (0.9%)	4 (0.6%)
*N* missing	21	8	14

**Fig. 2:**
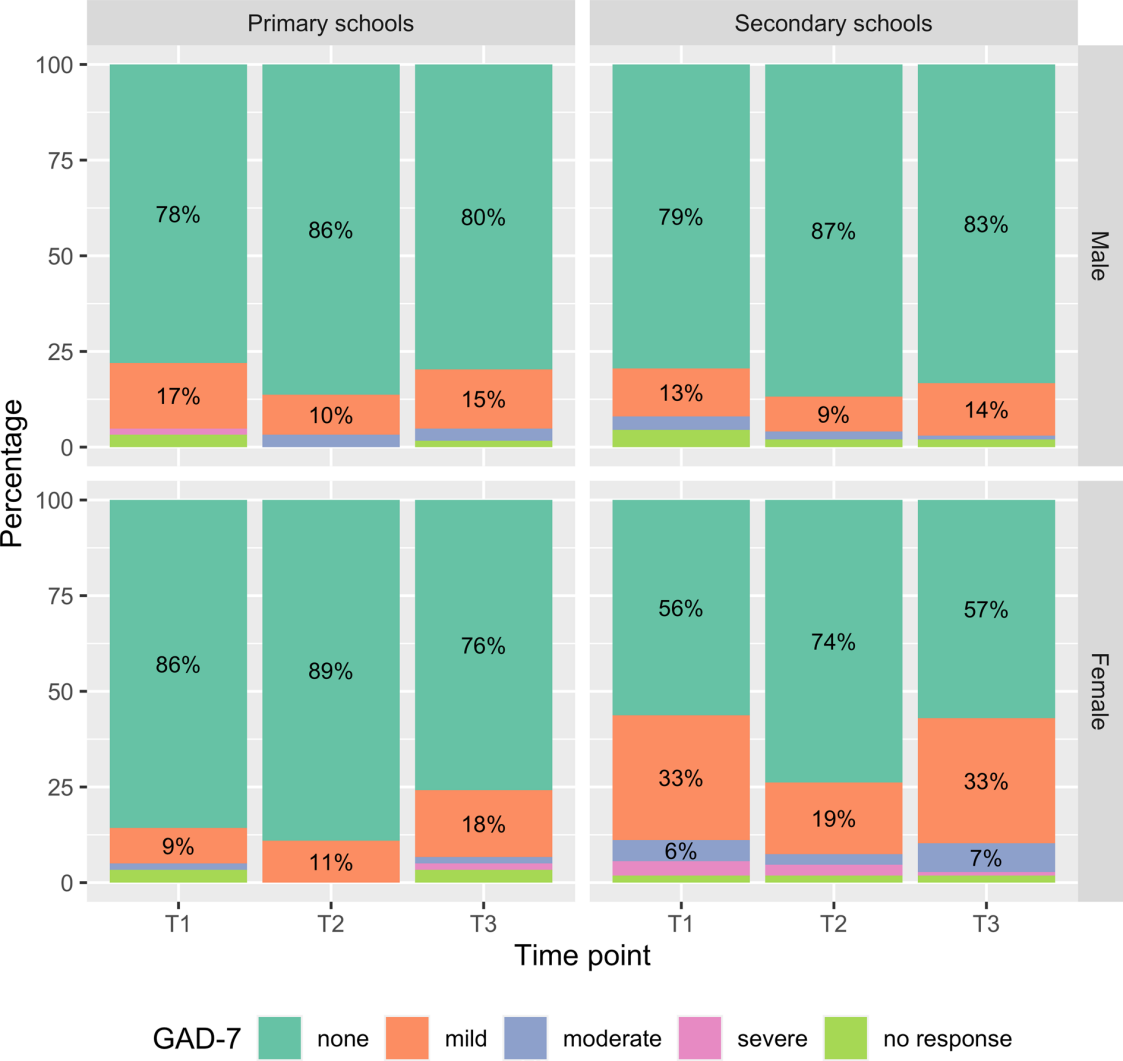
GAD-7 anxiety symptoms stratified by school form (primary/secondary) and sex (male/female) per time point in the inverse probability-weighted population

Stratifying these results by sex and school type showed that females, especially in secondary schools, were much more affected across all time points (Fig. 2). At T1, 43% of females in secondary schools reported anxiety symptoms (33% mild; 6% moderate; 4% severe). In comparison, among male secondary students, 17% reported anxiety symptoms (13% mild; 4% moderate; no severe symptoms).

These patterns are also apparent from the logistic regression models (Table 3) which show that at T2, girls had 1.75 times the odds (95% CI 1.14, 2.69) to experience any anxiety symptoms compared to boys, rising to 2.65 times the odds at T3 (95% CI 1.82, 3.85).

Similarly, children in secondary schools had significantly higher odds ratios for reporting anxiety compared to their peers in primary school in the range of 1.60–2.36 per time point. This is also reflected in an effect of age, which shows that the odds ratio for anxiety was between 1.18 and 1.24 for a one-year increase in age. Whereas the school district SES did not seem to influence the odds for experiencing the outcome, other household characteristics did: lower household income (OR of 1.86 at T2 and 1.46 at T3), as well as lower household education (OR 1.94 at T2), were associated with higher odds for experiencing anxiety; for family migration background, there was a tendency for this observed at T3 (OR 1.46). With a steady increase in the proportion of vaccinated children from 4.3% at T1 up to 47% at T3, the odds of anxiety were generally higher for those vaccinated compared to children who were not. The odds ratio declined from 1.96 to 1.25 as more children received the vaccine. While self-reported previous SARS-CoV-2 infections were relatively rare (5–7%) and did not increase much across time points, those children who reported a prior infection showed 0.14–0.26 times the odds of having anxiety symptoms compared with children who had not experienced SARS-CoV-2 infection at T3 and T1 respectively (Table 3).

**Table 3:** Odds ratios for binary GAD-7 outcome in the inverse probability-weighted population

	OR [CI]	T1	T2	T3
Sex	Female vs. male	**2.15** **[1.48, 3.13]**	**1.75** **[1.14, 2.69]**	**2.65** **[1.82, 3.85]**
Age	One year increase	**1.24** **[1.14, 1.35]**	**1.18** **[1.07, 1.31]**	**1.21** **[1.11, 1.32]**
School	Secondary vs. primary^a^	**2.36** **[1.56, 3.57]**	**1.60** **[1.01, 2.53]**	**1.62** **[1.10, 2.37]**
School district SES	Middle vs. high	0.88 [0.56, 1.36]	1.38 [0.85, 2.23]	1.20 [0.79, 1.84]
	Low vs. high	0.86 [0.52, 1.42]	0.89 [0.48, 1.65]	1.02 [0.62, 1.68]
SARS-CoV-2 Vaccination	Any vs. none	1.96 [0.89, 4.32]	**1.64** **[1.02, 2.62]**	1.25 [0.87, 1.78]
SARS-CoV-2 infection	Yes vs. no	**0.18** **[0.04, 0.78]**	0.26 [0.06, 1.07]	**0.14** **[0.03, 0.57]**
Household income	Lower vs. higher	1.29 [0.90, 1.86]	**1.68** **[1.09, 2.58]**	**1.46** **[1.02, 2.08]**
Household education	Secondary vs. higher	1.56 [0.93, 2.62]	**1.94** **[1.05, 3.57]**	1.49 [0.90, 2.46]
Family migration background	Yes vs. no	1.38 [0.87, 2.18]	1.09 [0.66, 1.81]	1.46 [0.97, 2.22]

Significant ORs presented in bold

* CI* confidence interval

^a^Age range is 6–14 years for primary schools and 10–19 years for secondary schools

## Discussion

Our study assessed anxiety symptoms within a cohort of German students from public primary and secondary schools at three time points within the later COVID-19 pandemic stage. In June 2021, at the end of an inconsistent peri-pandemic school term with varying pandemic developments and disrupted school routine, one in four students enrolled in our study reported anxiety symptoms. Subsequent time points revealed a slightly lower proportion directly after a six-week vacation in August 2021 with 16%, rising again to 26% by September 2021. Although a few studies from varying global contexts and pandemic phases found as much as 40% prevalence of anxiety symptoms in children and adolescents [18, 22–25], most research, yet often from earlier pandemic stages, measured similar proportions of anxiety to ours ranging from 5 to 25% among school children or university students [23, 26–29].

Data from the previous BECOSS 1 phase among a different group of Berlin school children had shown slowly increasing anxiety proportions over time, with about 25% of children and adolescents being affected in June and November 2020, reaching 39% in February 2021 during a 6-weeks lockdown and slightly declining to 34% in March 2021 after the lockdown [15]. Our observed anxiety frequency of 16–26% later in 2021 appears as a logical continuation of this development, with a pandemic crisis slowly stabilizing after a first peak, and children and adolescents mentally adapting to the perceived new normal state. The decreasing prevalence of anxiety over time and the presumed habituation or adaptation effect have previously been described among adult populations during the COVID-19 pandemic [30–32].

In accordance with our results, the population-based COPSY-study reported decreasing anxiety symptoms in German children in the later pandemic stage, with 26% anxiety symptoms in September 2021 compared to 30% in January 2021; still, all observed levels considerably exceeded children's pre-pandemic anxiety level of 15% measured in the German BELLA study between 2014 and 2017 [22, 26]. Other German pre-pandemic data from 2014 showed that based on parent-reported ICD-10 classification of children aged 7–17 years, 6% fulfilled the criteria of GAD and 12% scored above the anxiety cut-off of the SCARED-5 tool [33]. The fact that presence of GAD symptoms in September 2021 was still about twice as high compared to pre-pandemic levels is an alarming demonstration of the long-lasting nature of the pandemic's toll on mental health of children and adolescents [34]. In this context, it is also important to acknowledge that mental health impairment does not necessarily run in parallel to pandemic peak events, as seen in the BECOSS 1 phase where children's anxiety was found to be highest during lockdown in March 2020 with relatively low incidence rates of SARS-CoV-2 infections [15].

Our BECOSS 2 data gives further reason to assume that mental health responses to the pandemic can continue to persist for a considerable amount of time regardless of infection dynamics [23, 35]. Notably, the majority of children and adolescents in our cohort reported mild GAD symptoms. However, mild anxiety disorders, if left unaddressed, can result in maladaptive coping strategies and a cascading effect on more severe mental health conditions, and thus demand early diagnosis and treatment [36–38]. Overall, the unprecedented presence of GAD symptoms in children and adolescents during the COVID-19 pandemic calls for an appropriate health policy response [22, 38], and the mental health system in Germany should find solutions to fast-track psychological support for school children with anxiety disorders or other mental health issues as the pandemic is continuing [39]. On the other hand, the role of schools as a place of social stability, but also as a place offering de facto psychological and mental health support for children and adolescents cannot be overemphasized [3] and should be a strong argument against disrupting school routines.

In our cohort, female and comparatively older students had higher levels of anxiety symptoms throughout all time points, in continuation of our BECOSS 1 results, where girls in secondary school were at almost threefold odds for anxiety compared with boys [15]. While other research has equally identified female sex to have higher anxiety levels during COVID-19 [18, 26, 28, 40], this observation is also present in the general population outside of the COVID-19 context [41]. Complex interactions of socio-behavioral, societal and environmental factors are likely involved in the association between female sex and GAD in school children [42]. Further targeted research is needed to unveil causal factors that contribute to this association; in addition, tangible gender-specific coping strategies should be focused on [26]. Lower household education level and income and, as a tendency, family migration background, were also associated with anxiety symptoms at one or more time points. Children from socioeconomically disadvantaged households have previously been identified of being particularly susceptible to detrimental mental health outcomes during the pandemic [21, 22]. Further in-depth analyses are required to disentangle the underlying mechanisms of socioeconomic factors, family structures and children's mental health.

Children with previously diagnosed SARS-CoV-2 infection had lower GAD scores. There are two possible interpretations of this finding. Firstly, children without anxiety symptoms may engage in behaviors possibly playing a role in increasing the chances for infection. Reversely, it is equally possible that children who went through an infection without complications are less anxious regarding reinfection. At the same time, children reporting anxiety symptoms were more likely to be vaccinated at the time of our data collection. In our longitudinal BECOSS 1 analysis, we found anxiety symptoms to be significantly linked with fear of COVID-19 infection [15], and it is plausible that individuals with less fear of infection did not take advantage of vaccination services at the point of data collection. As an extension to this, individuals who were prioritized for the first available vaccines were often those with preexisting medical conditions and an increased risk for complications in case of infection. Therefore, the small subgroup of children who got vaccinated first might have had increased levels of anxiety due to a potential risk status.

The strength of our study is that it is one of the few providing empirical data on child and adolescent psychological status from a later stage of the pandemic across three observation time points. As a limitation, there is a potential for selection bias, on one hand regarding possibly differing student populations in those schools who rejected participation compared to those who participated, and on the other hand regarding the school administrations' choice of classes to which participation was offered. We experienced non-response and loss to follow-up over time, however, IPW was applied to adjust for related non-participation and underlying selection bias. For some questions, non-response and negation were equally classified as negative answer which might have led to overestimation of the negative answer, as in the case of previous SARS-CoV-2 infection. As another limitation, it is impossible for this study to unravel mental health effects directly related to the pandemic itself, to pandemic responses like school closures, or to other external factors like the summer vacation.

In conclusion, anxiety levels in children and adolescents from Berlin schools were lower in the second COVID-19 year than at previous pandemic time points, but still about twice as high compared to pre-pandemic data, pointing to the long-term pandemic impact on children's mental health response. Our results should urge policymakers to pay particular attention to the mental health status of school children even as the pandemic situation may stabilize and eventually appear as a new normal state. Prevention and coping strategies should give special consideration to female and older school children and to those from socioeconomically less advantaged families. To better understand the potential interplay of such factors and develop meaningful psychological support, more targeted in-depth analyses are required.

## Data Availability

The datasets used and analyzed during the current study are available from the corresponding author on reasonable request.
